# Molecular Characterization and Pathogenicity of a Novel Soybean-Infecting Monopartite Geminivirus in China

**DOI:** 10.3390/v14020341

**Published:** 2022-02-08

**Authors:** Min Du, Yongzhi Wang, Cheng Chen, Xiaoyu Li, Runzi Feng, Xueping Zhou, Xiuling Yang

**Affiliations:** 1 State Key Laboratory for Biology of Plant Diseases and Insect Pests, Institute of Plant Protection, Chinese Academy of Agricultural Sciences, Beijing 100193, China; dumincaas@163.com (M.D.); yzwang@126.com (Y.W.); chenfungi@126.com (C.C.); runzifeng1997@163.com (R.F.); 2 Key Laboratory of Integrated Pest Management on Crops in Northeast, Ministry of Agriculture, Jilin Academy of Agricultural Sciences, Changchun 130033, China; lxyzsx@163.com; 3 Key Laboratory of Integrated Pest Management on Crops in Southwest, Institute of Plant Protection, Ministry of Agriculture, Sichuan Academy of Agricultural Sciences, Chengdu 610066, China; 4 State Key Laboratory of Rice Biology, Institute of Biotechnology, Zhejiang University, Hangzhou 310058, China

**Keywords:** geminivirus, soybean, recombination, ssDNA virus

## Abstract

Soybean is a major legume crop that plays an important role in food production, industrial production, and animal husbandry. Here, we characterize a novel soybean-infecting monopartite geminivirus identified in China. Analysis of the contigs *de novo* assembled from sequenced small interfering RNAs, followed by PCR, cloning, and sequencing, the complete viral genome was determined to be 2782 nucleotides. The genome contains the conserved nonanucleotide sequence, TAATATTAC and other sequence features typical of the family *Geminiviridae*, and encodes two and four open reading frames in the virion-sense and the complementary-sense strands, respectively. Genome-wide pairwise identity analysis revealed that the novel virus shares less than 65.6% identity with previously characterized geminiviruses. Phylogenetic and recombination analysis indicated that this virus was placed in a unique taxon within the family *Geminiviridae* and potentially arose from recombination. An infectious clone of this virus was further constructed and its infectivity was tested in different species of plants. Successful infection and characteristic symptoms were observed in *Glycine max*, *Nicotiana benthamiana*, *N. tabacum*, *N. glutinosa*, and *N. tabacum* cv. Samsun plants. Taken together, this virus represents a member of an unclassified genus of the family *Geminiviridae*, for which the name soybean yellow leaf curl virus is proposed.

## 1. Introduction

*Geminiviridae* is a family of circular single-stranded DNA (ssDNA) viruses that constitute the largest family of known plant-infecting viruses and have caused many devastating diseases in a wide range of crops, vegetables, and ornamental plants worldwide [1,2]. With the development of a high-throughput sequencing-based virus discovery technique, an increasing number of geminivirus genome sequences have been available in the last couple of years. Novel highly divergent geminiviruses, such as tomato apical leaf curl virus, mulberry mosaic dwarf-associated virus, apple geminivirus, and passion fruit chlorotic mottle virus, have been identified from both economically relevant crops and woody perennials [3,4,5,6]. Currently, geminiviruses encompass 520 species (http://talk.ictvonline.org/taxonomy/, accessed on 10 November 2021) and are classified into 14 genera based on genome structure, insect vector, host range, and genome-wide pairwise sequence identity (*Becurtovirus*, *Begomovirus*, *Capulavirus*, *Curtovirus*, *Eragrovirus*, *Grablovirus*, *Mastrevirus*, *Topocuvirus*, *Turncurtovirus*, *Maldovirus*, *Opunvirus*, *Citlodavirus*, *Topilevirus*, *Mulcrilevirus*) [7,8]. Such a high number of species can be attributed to many factors, including the evolution of variants via mutation, recombination, and pseudo-recombination, acquisition of new components and satellites, high prevalence of insect vectors, global climate changes, and human activities [9].

The great majority of geminiviruses have a monopartite ssDNA genome that is encapsidated into twinned icosahedral particles. Their genomic components vary between 2.5 and 3.2 kb in length, and encode four to seven proteins through bidirectional transcription and partially overlapping open reading frames (ORFs). Members of the largest genus, *Begomovirus*, have either monopartite or bipartite ssDNA component(s). The replication-associated protein (Rep) and the capsid protein (CP), encoded by the C1 and V1 ORF, are the only two proteins that are conserved across all geminiviruses. Three other proteins, the transactivation protein (TrAP) encoded by C2, replication enhancer protein (REn) encoded by C3, and the C4 protein, are positionally conserved across the genera *Begomovirus*, *Curtovirus, Topocuvirus*, *Turncurtovirus*, *Maldovirus*, and *Opunvirus*, but the functions of these proteins vary and possibly are diverse from different lineages of geminiviruses.

Soybean (*Glycine max*) is a major legume crop that is known for its rich protein and oil content. It plays an important role in food production, industrial production, and animal husbandry. Similar to many agriculturally important crops, soybean plants are constantly attacked by both abiotic and biotic stresses and a handful of soybean viral diseases have been documented [10]. Viruses such as soybean mosaic virus, soybean dwarf virus, bean pod mottle virus, cowpea mild mottle virus, and alfalfa mosaic virus are the major reported soybean-infecting viruses that have posed a serious economic threat to soybean production, typically causing 10–30% yield losses under natural field conditions. Fifty percent to eighty percent yield losses were also reported for severe outbreaks [11]. In this study, we conducted a high-throughput sequencing of small RNAs isolated from diseased soybean leaf samples, collected from Jilin province of China. We identified a novel highly divergent geminivirus and demonstrated the infectivity of the new virus in its natural host and experimental hosts. The name soybean yellow leaf curl virus (SbYLCV) is proposed and could be considered as a type member of a new putative genus of the *Geminiviridae* family.

## 2. Materials and Methods

### 2.1. Plant Materials and Growth Conditions

Soybean plants showing yellowing and dwarf symptoms were collected from Jilin province of China in 2018. *Glycine max* cv. Williams 82, *Nicotiana benthamiana*, *N. tabacum*, *N. tabacum* cv. Samsun NN, *N. tabacum* cv. Samsun. nn, *N. glutinosa*, and *Solanum lycopersicum* cv. Moneymaker plants used for agrobacterium-mediated inoculation were grown in an insect-free growth room at 25 °C with a 16:8 h (light/dark) photoperiod.

### 2.2. Small RNA-Based Deep Sequencing

Total RNA was extracted from 1.0 μg of symptomatic soybean plant leaves using TRIzol reagent following the manufacturer’s protocols (Invitrogen, Carlsbad, CA, USA). Small RNAs (sRNAs) were purified from the extracted total RNA using Small RNA Sample Pre Kit (Illumina, San Diego, CA, USA), which were sequentially ligated with 3′ and 5′ adapters, reverse transcribed to complementary DNA (cDNA), and PCR amplified as described [12]. The resulting sRNA cDNA library was subjected to deep sequencing on an Illumina HiSeq^TM^ 2000 platform (Novogene, Beijing, China). After the removal of adapter sequences and low-quality reads, high-quality clean reads were assembled using the Velvet program [13]. The assembled contigs were subsequently screened for candidate virus(es) by BLASTn on the GenBank Virus Reference Sequence Database.

### 2.3. DNA Extraction and Amplification of the Full-Length Viral Genome

Total DNA was extracted from plant leaves using a cetyltrimethyl ammonium bromide (CTAB)-based extraction procedure, as described [14]. Genomic DNA was amplified by rolling circle amplification (RCA) with Templiphi^TM^ Kit (GE Healthcare, Sugar Notch, PA, USA) following the manufacturer’s protocols. RCA products were diluted five-fold and used for polymerase chain reaction (PCR) amplification using a pair of adjacent primers, Virus-F1/Virus-R1 (Table S1), designed based on assembled viral contigs identified by BLAST analysis. PCR was conducted with the TransStart FastPfu DNA polymerase (TransGen Biotech, Beijing, China) following the manufacturer’s instructions. The PCR program was set as follows: initial denaturation at 95 °C for 2 min; 35 cycles of denaturation at 95 °C for 20 s, annealing at 55 °C for 20 s, and amplification at 72 °C for 2 min; and a final extension at 72 °C for 5 min. The expected 2.8-kb PCR product was confirmed by electrophoresis and gel-purified using the E.Z.N.A. Gel Extraction Kit (Omega, Norcross, GA, USA). The purified fragments were cloned into the pEASY-T-Blunt vector (TransGen) and transformed into competent *Escherichia coli* DH5α cells. The recombinant clones were screened and sequenced with universal primer pairs, M13F/M13R, and walking primers via Sanger sequencing (TsingKe Biotech Co., Beijing, China). Based on the full-length genome sequence, the other pair of adjacent primers, Virus-F2/Virus-R2 (Table S1), were used to amplify the full-length fragment via a similar strategy.

### 2.4. Genome Characterization

Sequences were edited and assembled using the Lasegene 7 Software (DNASTAR Inc., Madison, WI, USA). Open reading frames (ORFs) encoded by the complete viral genome were predicted using ORF Finder (http://www.ncbi.nlm.nih.gov/gorf/gorf.html, accessed on 27 November 2019). The reconstructed genome sequences were subjected to the BLASTn algorithm (https://blast.ncbi.nlm.nih.gov/Blast.cgi, accessed on 27 November 2019) to identify which of the sequences were most related to.

### 2.5. Pairwise Distances, Phylogenetic, and Recombination Analysis

The representative complete genome sequences of the 14 genera of the family *Geminiviridae* were retrieved from the GenBank database and used for delimiting species boundaries. The amino acid sequences of inferred capsid protein (CP) and replication-associated protein (Rep) were selected from the complete viral genomes. Pairwise nucleotide sequence comparisons for the full-length genome and amino acid sequence comparisons for Rep and CP were conducted using the Sequence Demarcation Tool (SDT) v1.2 [15]. Alignment of the nucleotide or amino acid sequences of target sequences was performed using Clustal W and the neighbor-joining phylogenetic trees were constructed with 1000 bootstrap replicates using the MEGA software version 5.0 [16]. Recombination analysis was carried out using the recombination detection program RDP4 [17]. Sequences were aligned in Muscle in MEGA5 and exported to the RDP4 program to test potential recombination events, using more than one method for authenticity.

### 2.6. Infectivity Assays

To generate the infectious clone of SbYLCV, two-tandem repeats of SbYLCV were cloned into the pBinPLUS vector using a one-step assembly strategy [18]. In brief, two pairs of primers were designed based on the complete sequence of SbYLCV and the pBinPLUS vector, with 15 bp overlapping nucleotides at fragment ends. Primer sets, SbYLCV-*Sal*I-1F/SbYLCV-1R and SbYLCV-2F/SbYLCV-*EcoR*I-2R (Table S1) were used to amplify individual full-length fragments of SbYLCV. PCR reactions were conducted with TransStart FastPfu high-fidelity DNA polymerase (TransGen) with DNA of the Jilin isolate as a template. The pBinPLUS vector was digested with FastDigest restriction enzymes, *Sal*I and *EcoR*I. PCR products and the linearized pBinPLUS vector were gel-purified with E.Z.N.A Gel Extraction Kit (Omega Bio-Tek, Norcross, GA, USA). The two overlapping full-length fragments of SbYLCV were seamlessly assembled to the linearized pBinPLUS vector using the In-Fusion^®^ HD Cloning Kit according to the manufacturer’s instructions (Takara, Tokyo, Japan). The positive clones were screened by colony PCR and plasmids were extracted and digested with the restriction enzyme, *EcoR*I to verify the correct insertion of two tandem repeats. Three independent clones were further sequenced to ensure that no mutations were introduced. The resultant recombinant clone, pBinPLUS-SbYLCV 2A was transformed into *Agrobacterium tumefaciens* EHA105 by electroporation.

### 2.7. Agroinoculation of Plants

Assays regarding the infectivity of the infectious clone of SbYLCV in tested plants were conducted, as previously described [19]. *A. tumefaciens* clones carrying the two tandem repeats of SbYLCV were cultured in LB broth with kanamycin (50 μg/mL) and rifampicin (50 μg/mL) overnight at 28 °C. After centrifugation at 4000× *g* for 10 min, the pelleted agrobacteria cells were resuspended to an optical density OD_600_ of 1.0 with infiltration buffer (10 mM MgCl_2_, 10 mM MES (pH 5.8) and 100 μM acetosyringone), and incubated at room temperature for 2–3 h before infiltration. To test the infectivity of the infectious clone of SbYLCV in solanaceous plants, plants at the 4–6 leaf stage were used for agroinoculation, as described [19]. For agroinoculation of *N. benthamiana*, *N. tabacum*, and *N. glutinosa*, resuspended agrobacteria cells were infiltrated into the abaxial surface of plant leaves using a 1 mL needleless syringe. For agroinoculation of tomato plants, the agrobacteria suspension was injected into the stems and abaxial leaves using a 1 mL fine syringe. For agroinoculation of soybean plants, cotyledon knots of two-day-old sprouted soybean seeds were injected with agrobacteria suspension using a 1 mL fine syringe. In all the experiments, plants inoculated with *A. tumefaciens* containing the empty vector, pBinPLUS were used as negative controls. Inoculated plants were placed in an insect-free greenhouse and visualized daily for symptom development. Photos were taken with a Canon 400D digital camera.

### 2.8. Southern Blot Assay

Nucleic acids were isolated from young leaves of inoculated plants at 30 days post-inoculation. Samples of total genomic DNA (20 μg) were electrophoresed in 1.5% agarose gel and stained with ethidium bromide to provide a loading control. After denaturation and neutralization, total DNA was transferred to Hybond N^+^ nylon membranes (GE Healthcare) by capillary transfer. Membranes were hybridized at 50 °C to digoxigenin-labeled probes specific to SbYLCV. After washing, the hybridization signals were detected using the anti-digoxigenin AP chemiluminescent substrate CSPD following the manufacturer’s instructions (Roche Diagnostics, Rotkreuz, Switzerland). The results were visualized with a chemiluminescence detection system (Tianneng, Shanghai, China).

## 3. Results

### 3.1. Discovery of a Novel Soybean-Infecting Monopartite Geminivirus

To identify possible viruses in the collected soybean samples, total RNA was extracted from symptomatic leaves and subjected to deep sequencing using the Illumina Hiseq2000 platform. A total of 20,380,853 clean reads were obtained after filtering the 20,914,978 raw reads by trimming low-quality reads, 5′- and 3′-adapter sequences, and poly A/T/C/G reads. *De novo* assembly of the small RNA reads using the Velvet program generated 999 long contigs. BLASTn searches of the long contigs against the GenBank nucleotide database revealed that the longest contig (347 nucleotides) was closely related to begomovirus C1, sharing the highest nucleotide identity (85%) with ageratum yellow vein virus isolate AFSP8d (accession number JN809826.1). Considering the circular nature of the geminivirus genome, adjacent primers Virus-F1/Virus-R1 were designed based on the longest contig to amplify the putative full-length geminivirus-like genome. Two symptomatic soybean leaf samples and one asymptomatic soybean sample were used for the detection of the candidate virus. Amplicons of approximately 3.0 kb in length were amplified from the two symptomatic soybean leaf samples, but not from the asymptomatic soybean leaves. Cloning and sequencing of the amplicons generated inserts of 2782 nucleotides (nts) in size. To reconfirm the circular characteristic of the soybean-infecting DNA molecule, another pair of adjacent primers, Virus-F2/Virus-R2, were designed based on the 2782 bp sequence. A band of about 3 kb was also successfully amplified from the symptomatic soybean leaves. Cloning and sequencing of the fragment revealed that the amplified 2782 bp sequence was identical to the sequence obtained by Virus-F1/Virus-R1, suggesting that the amplified 2782 bp sequence represents the full genome of a circular DNA virus. The whole genome sequence has been deposited in Genbank (accession number OL404964). BLASTn analysis, using the 2782 bp sequence as a query, showed that the region from 1615 to 2782 nt has a top similarity (83%) with tomato leaf curl Java virus-[Ageratum] (ToLCJV) (accession number AB162141.1), and the other region of about 1600 nt has no significant similarity with known sequences. Pairwise identity of the 2782 bp sequence with the complete genome sequence of ToLCJV revealed that they share 65.6% genome-wide pairwise identity, supporting the likelihood that the circular DNA isolated from the symptomatic soybean leaves represents a previously uncharacterized geminivirus.

To determine whether the identified soybean-infecting geminivirus is associated with a putative DNA-B component, an alphasatellite, or a betasatellite, the DNA sample obtained from the same diseased soybean plant was used as a template and PCR was performed using degenerate primer pairs, CR01/CR02 [20], UNA101/UNA102 [21], and β01/β02 [22], respectively. Consistent with the deep sequencing data, no amplified product was detected with any of the primer sets (data not shown). Taken together, the results suggest that the soybean-infecting DNA molecular is a monopartite geminivirus, for which the name soybean yellow leaf curl virus (SbYLCV) is tentatively proposed.

### 3.2. Genome Organization of SbYLCV

**Figure 1:**
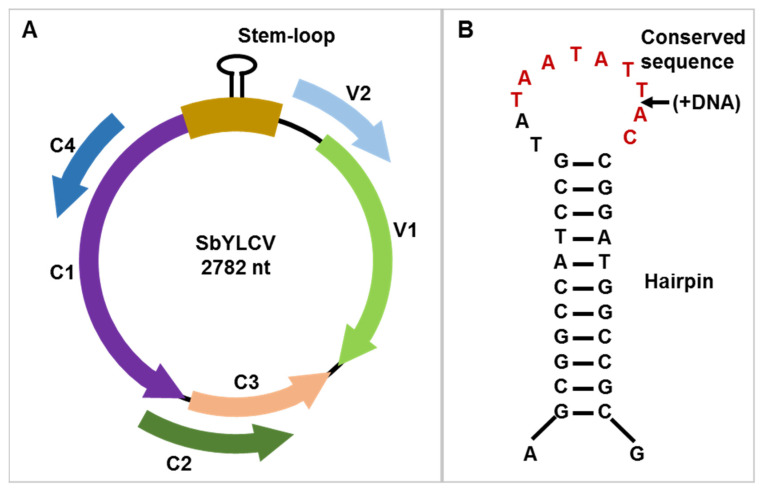
Genome organization of soybean yellow leaf curl virus (SbYLCV). (**A**) Schematic representation of SbYLCV genomic organization. Open reading frames encoded on the virion-sense (V) strand and complementary-sense (C) strand are denoted with different colors. (**B**) The stem-loop structure located within the intergenic region of SbYLCV. The conserved nonanucleotide sequence is shown in red color. The putative nick site within the nonanucleotide sequences is marked with an arrow.

By using the ORF finder (https://www.ncbi.nlm.nih.gov/orffinder/, accessed on 27 November 2019), six recognizable ORFs-encoding proteins composed by >96 amino acids were identified, considering both strands of the SbYLCV genome. The arrangement of the six ORFs within the SbYLCV genome resembles those described for monopartite begomoviruses, topocuviruses, turncurtoviruses, maldoviruses, and opunviruses, with two ORFs (V1 and V2) on the virion strand and four (C1, C2, C3, and C4) on the complementary strand (Figure 1). The virion and complementary strands of SbYLCV are separated by an intergenic region (IR) of 285 nts, which shares a conserved nonanucleotide motif, TAATATTAC, with most geminiviruses (Figure 1 and Table 1).

**Figure 2:**
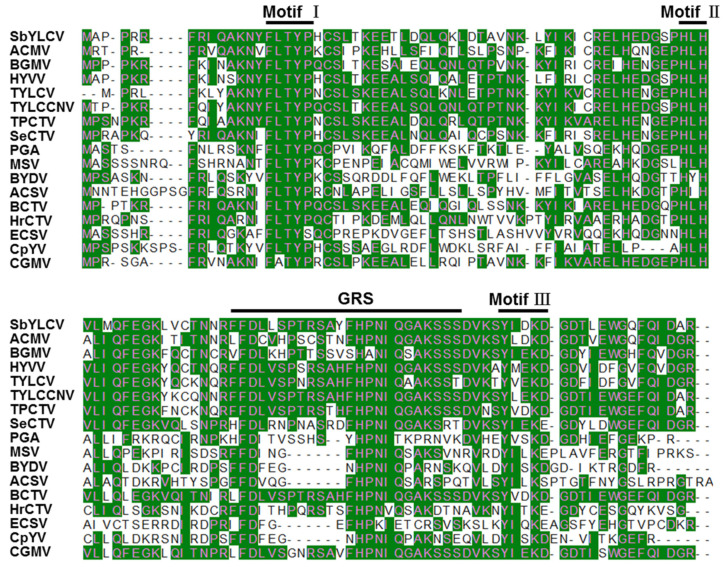
Alignment of the N-terminal sequences of the predicted SbYLCV Rep protein with those of selected geminiviruses. The conserved motifs I, II, III, and GRS are shown, as indicated. ACMV, African cassava mosaic virus; ACSV, *Axonopus compressus* streak virus; BCTV, beet curly top virus; BGMV, bean golden mosaic virus; BYDV, bean yellow dwarf virus; CGMV, cowpea golden mosaic virus; CpYV, chickpea yellows virus; ECSV, *Eragrostis curvula* streak virus; HrCTV, horseradish curly top virus; HYVV, honeysuckle yellow vein virus; MSV, maize streak virus; PGA, prunus geminivirus A; SeCTV, sesame curly top virus; TPCTV, tomato pseudo-curly top virus; TYLCCNV, tomato yellow leaf curl China virus; TYLCV, tomato yellow leaf curl virus.

BLASTP searches using the amino acid sequences of the six individual ORFs of SbYLCV revealed that most of the predicted SbYLCV proteins are highly divergent from those of known geminiviruses. The predicted V1 protein of SbYLCV shares a 32.37% of amino acid sequence identity with the coat protein (CP) of sweet potato symptomless virus 1. SMART analysis showed that the V1 protein was identified as a geminivirus CP. Although the predicted V2 protein had the highest amino acid sequence identity (41.89%) with the V2 protein of *Juncus maritimus*-associated virus, SMART analyses did not recognize any conserved domains in SbYLCV V2 (Table 1). On the contrary, the predicted C1 protein shares a high homology (87.72%) with the Rep protein of ageratum yellow vein China virus. Examination of the conserved domains by SMART analysis revealed that both the conserved catalytic and central domains of geminivirus Rep proteins were found in SbYLCV C1. A comparison of SbYLCV C1 to selected geminiviruses indicated that SbYLCV contains typical domains required for geminivirus rolling circle replication: motif I (FLTYP) is required for specific dsDNA binding, motif II (HLH) is involved in protein conformation and DNA cleavage, motif III (YI/xD/EKD) is related to DNA cleavage, and a geminivirus Rep sequence (GRS)-conserved motif is responsible for rolling circle replication initiation (Figure 2) [23,24,25]. The predicted C2 and C3 protein of SbYLCV shares 51.22% and 51.26% identity with those of *Juncus maritimus*-associated virus. Similar to C1, the predicted SbYLCV C4 protein displays less divergence and shares the highest amino acid sequence identity (82.29%) with that of ageratum yellow vein virus. Taking into account the nucleotide sequence divergence of the full-length genome and the amino acid sequence of the predicted proteins encoded by SbYLCV, SbYLCV identified in this study is distinct from currently classified geminiviruses and represents a novel species.

### 3.3. Phylogenetic Relationships

**Figure 3:**
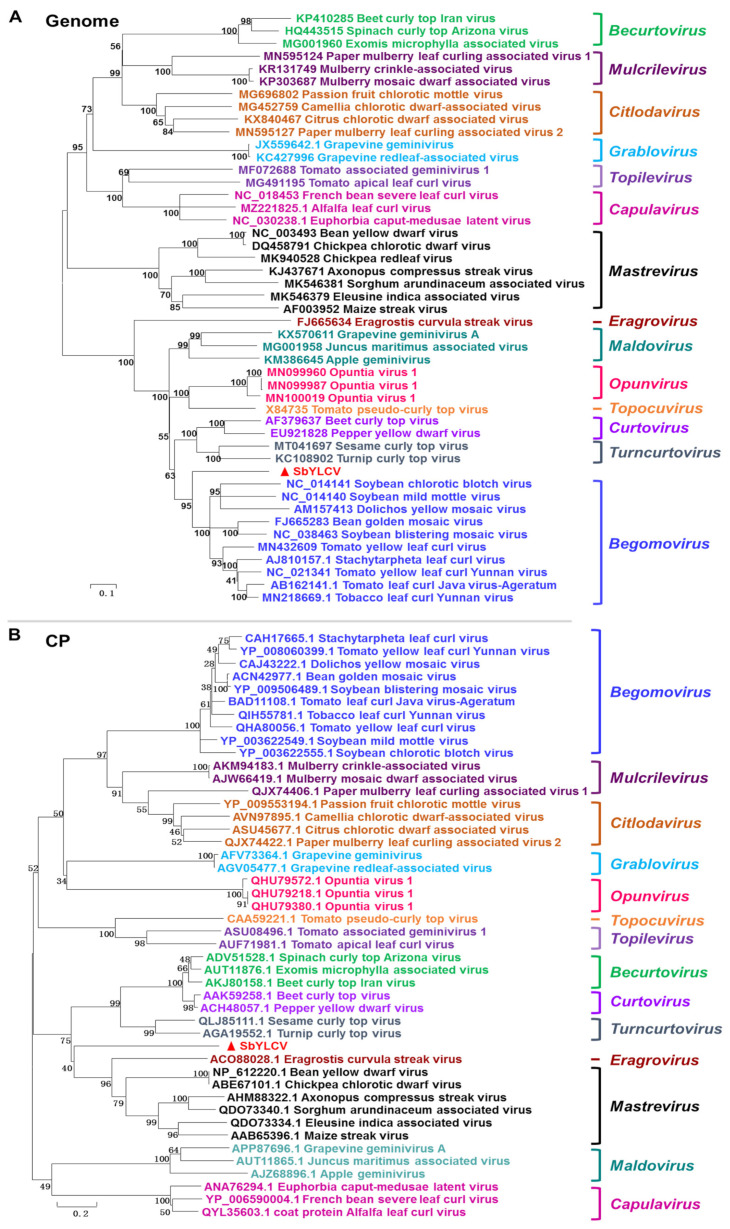
Phylogenetic relationships of SbYLCV and representative geminiviruses based on the nucleotide sequence of the full-length genome (**A**) and the amino acid sequence of the coat protein (CP) (**B**). The phylogenetic trees were constructed with MEGA 5.0 using the neighbor-joining method. The statistical significance of the branches was determined with a bootstrap of 1000 replicates.

To understand the evolutionary relationship of SbYLCV to other known geminiviruses, phylogenetic trees were individually constructed using the nucleotide sequences of the full-length genomes and the amino acid sequences of CP and Rep encoded by selected geminiviruses. The full-length genome-based phylogeny showed that SbYLCV does not group with geminiviruses belonging to any of the established 14 genera (Figure 3A). Pairwise distance analysis from the color-coded matrix plot revealed that SbYLCV shares 43~65.6% genome-wide pairwise identities with other geminiviruses (Figure S1).

Similarly, phylogenetic analysis of the predicted SbYLCV CP amino acid sequences with those of representative geminiviruses revealed that SbYLCV CP forms a distinct clade and is most closely related to mastreviruses and the *Eragrostis curvula* streak virus (ECSV) (Figure 3B). Comparison of protein sequences revealed that the predicted CP protein of SbYLCV share <33% amino acid sequence similarity with the CP of other geminiviruses (Figure S2).

When the phylogenetic tree was generated using SbYLCV Rep amino acid sequences together with the Rep of other representative geminiviruses, it was inferred that the SbYLCV Rep sequence groups together with the Rep of begomoviruses (Figure S3), an observation that was consistent with the high sequence identities of the pairwise distance analysis (Figure S4).

### 3.4. Recombination Analysis

**Figure 4:**
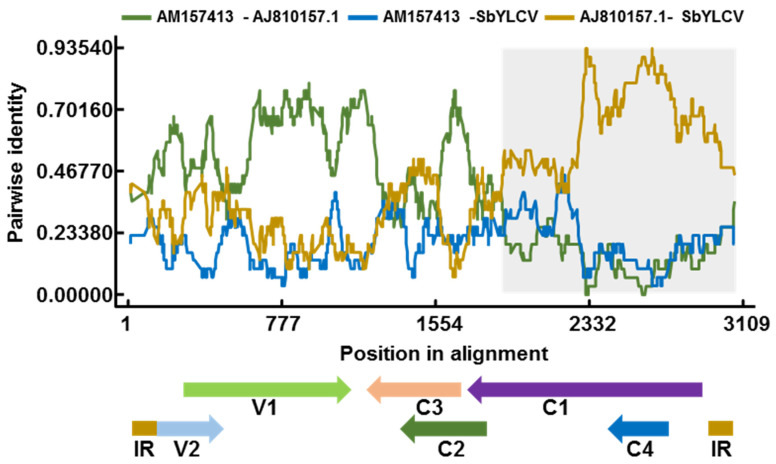
Recombination analysis of SbYLCV. The recombinant event was detected using the Recombination Detection Program RDP4. The brown line indicates the pairwise identity between the minor parent (Stachytarpheta leaf curl virus, AJ810157.1) and SbYLCV. The grey region represents the location of predicted breakpoints. The displayed linearized genome organization of SbYLCV shows the position in the alignment.

As recombination plays a pivotal role in driving the emergence and evolution of geminiviruses [26], the RDP software was used to examine the SbYLCV sequence for evidence of recombination. One recombination event was detected with a high degree of confidence by six recombination detection methods: RDP (average *p* value, 2.66 × 10^−24^), GENECONV (average *p* value, 3.79 × 10^−2^°), Bootscan (average *p* value, 2.79 × 10^−21^), MaxChi (average *p* value, 1.04 × 10^−17^), Chimera (average *p* value, 3.05 × 10^−4^), and SiSscan (average *p* value, 2.79 × 10^−23^). The breakpoints for this predicted event ranged from nucleotides 1674 to 2760. The sequence of stachytarpheta leaf curl virus (accession number AJ810157.1) in the analyzed dataset was implicated as the putative minor parent, and those of Dolichos yellow mosaic virus (accession number AM157413) and an unknown virus, resemble the putative major parent (Figure 4). Hence, SbYLCV is a novel virus arising from recombination.

### 3.5. Infectivity of the Infectious Clone of SbYLCV

**Figure 5:**
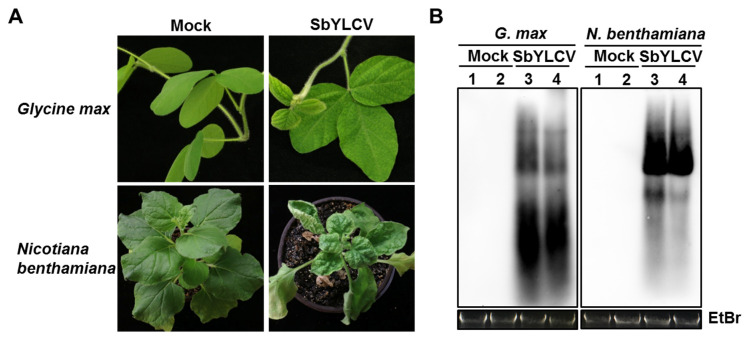
Systemic infection of SbYLCV in *Glycine max* and *Nicotiana benthamiana* plants. (**A**) Characteristic symptoms induced by SbYLCV. Plants were agroinoculated with the pBinPLUS vector (mock) or the infectious clone of SbYLCV, as indicated. Photos were taken at 30 days post-inoculation (dpi). (**B**) Southern blot hybridization analysis of SbYLCV DNA with a specific DIG-labelled DNA probe. Total DNA was extracted from systemic leaves of SbYLCV-infected and mock-inoculated plants, as indicated. Ethidium bromide staining of total DNA was used as loading controls.

To test the infectivity of SbYLCV, two-tandem repeats of SbYLCV full-length fragments were cloned into the binary vector pBinPLUS to generate pBin-SbYLCV-2A. Infectivity tests were first performed in soybean (natural host) and *N. benthamiana* plants, the widely used model plant for studying the virus–host interaction. At 14 days post-agroinoculation (dpi), five of the 52 soybean plants inoculated with pBin-SbYLCV-2A displayed leaf curling and yellowing symptoms, which were visually different from that inoculated with the empty vector pBinPLUS (mock treatment; Figure 5A, Table 2). Leaf curling and stunting symptoms were also observed in all the *N. benthamiana* plants (*n* = 34) agroinfiltrated with pBin-SbYLCV-2A, but not in those agroinfiltrated with the empty vector pBinPLUS (Figure 5A, Table 2). Southern blot hybridization, using a specific probe for SbYLCV, demonstrated the systemic infection and efficient replication of SbYLCV in both soybean and *N. benthamiana* plants (Figure 5B), indicating that the developed infectious clone of SbYLCV is infectious in both soybean and *N. benthamiana* plants.

**Figure 6:**
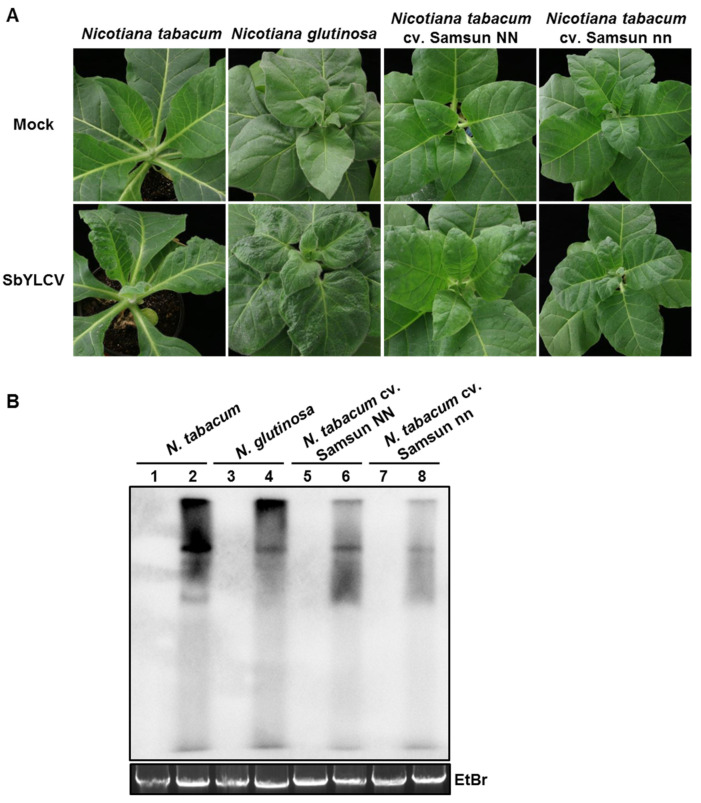
Systemic infection of SbYLCV in *Nicotiana tabacum*, *N. glutinosa*, *N. tabacum* cv. Samsun NN, and *N. tabacum* cv. Samsun nn plants. (**A**) Characteristic symptoms induced by SbYLCV. Plants were agroinoculated with the pBinPLUS vector (mock) or the infectious clone of SbYLCV, as indicated. Photos were taken at 30 days post-inoculation (dpi). (**B**) Southern blot hybridization analysis of SbYLCV DNA with a specific DIG-labelled DNA probe. Total DNA was extracted from systemic leaves of SbYLCV-infected and mock-inoculated plants, as indicated. Lanes 1, 3, 5, and 7 represent total DNA extracted from plants inoculated with the pBinPLUS vector; lanes 2, 4, 6, and 8 represent total DNA extracted from plants inoculated with the infectious clone of SbYLCV. Ethidium bromide staining of total DNA was used as a loading control.

As many geminiviruses can infect *Solanaceae* crops, the infectivity of SbYLCV was further assessed on four other plant species of the family *Solanaceae*. Agroinoculated plants were closely monitored for symptom development. At 21 dpi, a typical leaf curling symptom was visible in the systemic leaves of *N. tabacum*, *N. tabacum* cv. Samsun, and *N. glutinosa* plants agroinfiltrated with pBin-SbYLCV-2A (Figure 6A, Table 2). Detection of viral DNA using Southern blot hybridization inferred the accumulation of viral DNA in these symptomatic leaves (Figure 6B). In contrast, no symptoms were observed in tomato plants agroinoculated with pBin-SbYLCV-2A. When PCR was used to detect SbYLCV, negative results were found in the systemic leaves of agroinoculated tomato plants (Table 2). These results collectively suggest that the infectious clone of SbYLCV is infectious in *N. tabacum*, *N. tabacum* cv. Samsun, and *N. glutinosa* plants, but not in tomato plants.

## 4. Discussion

Geminiviruses represent a threat to agriculture worldwide. In the last decade, viral metagenomic studies have contributed to the discovery of a growing number of geminiviruses. Here, we identified and characterized a novel monopartite geminivirus infecting soybean in China.

The arrangement of the six predicted ORFs encoded by the novel circular DNA virus is similar to that of monopartite begomoviruses, topocuviruses, turncurtoviruses, maldoviruses, and opunviruses. It also shares several common features with known geminiviruses, including the stem-loop structure and the conserved nanonucleotide, TAATATTAC, within the intergenic region and several critical motifs in the Rep ORF. However, pairwise sequence identity comparison and phylogenetic analyses of the full-length genome showed that this novel DNA virus is distinct from the established 14 genera of the *Geminiviridae* family, which only shares the highest sequence similarity (65.6%) with ToLCJV. These observations suggest that this novel circular DNA virus represents a highly divergent species of a putative new genus of the *Geminiviridae* family, for which the name soybean yellow leaf curl virus (SbYLCV) is proposed.

Sequence analysis of the predicted SbYLCV proteins revealed that the Rep and C4 ORFs are closely related to those of begomoviruses, whereas the predicted V1, V2, C2, and C3 proteins share less than 52% amino acid sequence identity with those of other reported geminiviruses. This indicates that SbYLCV is a recombination-prone virus, which was further supported by six recombination detection methods implemented in the RDP software. Recombination is an important force driving geminivirus evolution, which can provide a strong selective advantage in virus species at the species and genus level. Previously, numerous begomoviruses have been reported to arise from recombination and the resulting recombinants often have an increased fitness or pathogenesis. The acquisition of a more virulent C4 arisen by recombination results in a more virulent begomovirus that does not require a betasatellite for symptom induction [27]. It would be interesting to understand whether novel roles have likely been acquired by SbYLCV during recombination.

Betasatellites are small circle ssDNA molecules that are frequently found in association with many begomoviruses and occasionally with mastreviruses [1]. In line with the deep sequencing data, no DNA B component, alphasatellite, or betasatellite was detected in the collected soybean sample by PCR. Infectivity tests with the constructed infectious clone further revealed the successful infection of SbYLCV in both the natural host soybean and the selected experimental hosts, conclusively demonstrating that SbYLCV is a monopartite geminivirus. Previously, betasatellites were proved to be replicated promiscuously by distinct geminiviruses and could be modified as a valuable versatile vector to suppress gene expression in plants [28,29,30]. It would be interesting to test whether the SbYLCV identified in this study could support the replication of different satellites, which would provide a valuable tool to study gene function in soybean plants.

Previous studies have shown that some functions of positional homologs such as Rep are conserved across geminivirus species and genera, whereas other geminivirus-encoded proteins may display a diversity of functions in different species [31]. For example, several independent functions have been ascribed to C4/AC4 and C2/AC2 of different geminivirus species [32,33]. Recent years have witnessed an unprecedented understanding of the begomovirus protein structure and function; however, very limited information is available for the newly identified geminivirus species. Although SbYLCV has a similar genome organization to monopartite begomoviruses, topocuviruses, turncurtoviruses, maldoviruses, and opunviruses, the function of SbYLCV-encoded proteins remains to be explored. It is noteworthy that we applied the 10 kDa threshold in the prediction of SbYLCV-encoded proteins, as shown in previous studies. A recent study showed that geminiviruses also encode small additional ORFs (size range 5–10 kDa) with specific subcellular localization and functions [34]. Additional ORF was also identified in the viral sense of the betasatellite genome and was demonstrated to have a critical role in virus infection [35]. These findings demand an exploration of small proteins in SbYLCV and an understanding of their roles in the infection cycle of SbYLCV.

Insect vectors play significant roles in geminivirus spread and evolution in nature, and CP is the only geminiviral protein that is involved in geminivirus transmission. The specificity between CPs with their insect vectors reflects an evolutionary relationship between geminiviruses and their transmission vector. A phylogenetic analysis of the predicted amino acid sequence of the CP of SbYLCV with the CP of geminiviruses representing all the 14 genera of the family *Geminiviridae* revealed that SbYLCV forms a distinct clade, but was most closely related to the CP of mastreviruses and ECSV. Mastreviruses are known to be transmitted by leafhoppers. Although the insect vector responsible for SbYLCV transmission in the field is scanty at this stage, the availability of the infectious clone developed here and their successful infection on soybean and other experimental hosts allow us to address this issue in the near future.

In summary, we have detected and characterized a novel soybean-infecting monopartite geminivirus in China. This novel geminivirus can replicate and establish a successful infection in inoculated soybean plants and five experimental hosts. These findings provide useful tools for the understanding of plant and geminivirus interactions and the selection of resistant soybean cultivars, which will be beneficial to the development of management strategies for disease control.

## Figures and Tables

**Table 1:** ORFs and encoded proteins in the genome of soybean yellow leaf curl virus.

ORF	Nucleotide Coordinates	Orientation	No. Amino Acids	*M*_r_ (kDa)	BLASTP (E-Value)	Predicted Domains (SMART)	Amino Acid Coordinates of Predicted Domain
V1	263–1048	Sense	262	29.4	Coat protein of sweet potato symptomless virus 1	Geminivirus coat protein/nuclear export factor BR1 family (1.04E-10)	5–259
V2	139–450	Sense	129	11.9	V2 of Juncus maritimus associated virus	-	-
C1	2637–1552	Complementary	362	41.3	Rep of ageratum yellow vein China virus	Geminivirus Rep catalytic domain (3.04E-62)	7–119
						Geminivirus rep protein central domain (5.73E-35)	126–230
C2	1649–1233	Complementary	139	15.4	C2 of Juncus maritimus associated virus	Geminivirus AL2 protein (3.18E-20)	1–135
C3	1537–1082	Complementary	152	17.6	C3 of Juncus maritimus associated virus	Geminivirus AL3 protein (4.13E-35)	18–132
C4	2480–2190	Complementary	97	10.5	C4 of ageratum yellow vein virus	Geminivirus C4 protein (1.72E-35)	1–84

**Table 2:** The infectivity analysis of soybean yellow leaf curl virus.

Plants	No. of Plants Infiltrated	No. of Plants Systemically Infected	Infection Efficiency (%)	Symptoms
*Nicotiana benthamiana*	34	34	100%	Leaf curling
*Glycine max*	52	5	9.6%	Leaf curling, yellowing
*Solanum lycopersicum*	15	0	0	NA
*Nicotiana tabacum*	16	13	81.3%	Leaf curling
*Nicotiana tabacum* cv. Samsun NN	10	7	70%	Leaf curling
*Nicotiana tabacum* cv. Samsun nn	10	3	33%	Leaf curling
*Nicotiana glutinosa*	10	2	20%	Leaf curling

## Data Availability

Not applicable.

## Supplementary Materials

The following is available online at https://www.mdpi.com/article/10.3390/v14020341/s1, Table S1. Sequences of primers used in this study; Figure S1. Matrix plot showing the pairwise identity of the complete genome sequences of SbYLCV and representative geminiviruses using the Sequence Demarcation Tool (SDT) software; Figure S2. Matrix plot showing the pairwise identity of the amino acid sequences of SbYLCV CP and the CP of representative geminiviruses using the SDT software; Figure S3. Neighbor-joining phylogeny tree constructed based on the amino acid sequences of SbYLCV Rep and the Rep of the representative geminiviruses. The statistical significance of the branches was estimated with a bootstrap of 1000 replicates; Figure S4. Matrix plot showing the pairwise identity of the amino acid sequences of SbYLCV Rep and the Rep of representative geminiviruses.
