# Supplementary material for: Molecular Characterization and Pathogenicity of a Novel Soybean-Infecting Monopartite Geminivirus in China
Source: Viruses. 2022 Feb 8;14(2):341. doi: 10.3390/v14020341 (PMC8877103; doi:10.3390/v14020341)

Table S1. Sequences of primers used in this study

Primer name	Primer sequences (5’-3’)
Virus-F1	CATTCCTCAAGTTCTTCCGG
Virus-R1	GAATGGATGTCAGAGAACGTCA
Virus-F2	CTAAATCAAGTTACATAGCCGGATC
Virus-R2	TTTAGCTGACTGTATAATGGAATTAGAATA
β01	GGTACCACTACGCTACGCAGCAGCC
β02	GGTACCTACCCTCCCAGGGGTACAC
CR01 (DNA-B)	CATATTTACRARWATGCCA
CR02 (DNA-B)	CARTGRTCKATCTTCATACA
UNA101(DNA1)	AAGCTTGCGACTATTGTATGAAAGAGG
UNA102(DNA1)	AAGCTTCGTCTGTCTTACGAGCTCGCTG
SbYLCV-*Sal*I-1F	CTTGCATGCCTGCAGGTCGACCATTCCTCAAGTTCTTCCGG
SYLCV-1R	TTGAGGAATGGATGTCAGAGAACGTCAAGG
SYLCV-2F	CTCTGACATCCATTCCTCAAGTTCTTCCGG
SYLCV-*Eco*RI-2R	GTAATTGTTAATTAAGAATTCGATGTCAGAGAACGTCAAGG

Figure S1. Matrix plot showing the pairwise identity of the complete genome sequences of SbYLCV and representative geminiviruses using the Sequence Demarcation Tool (SDT) software.

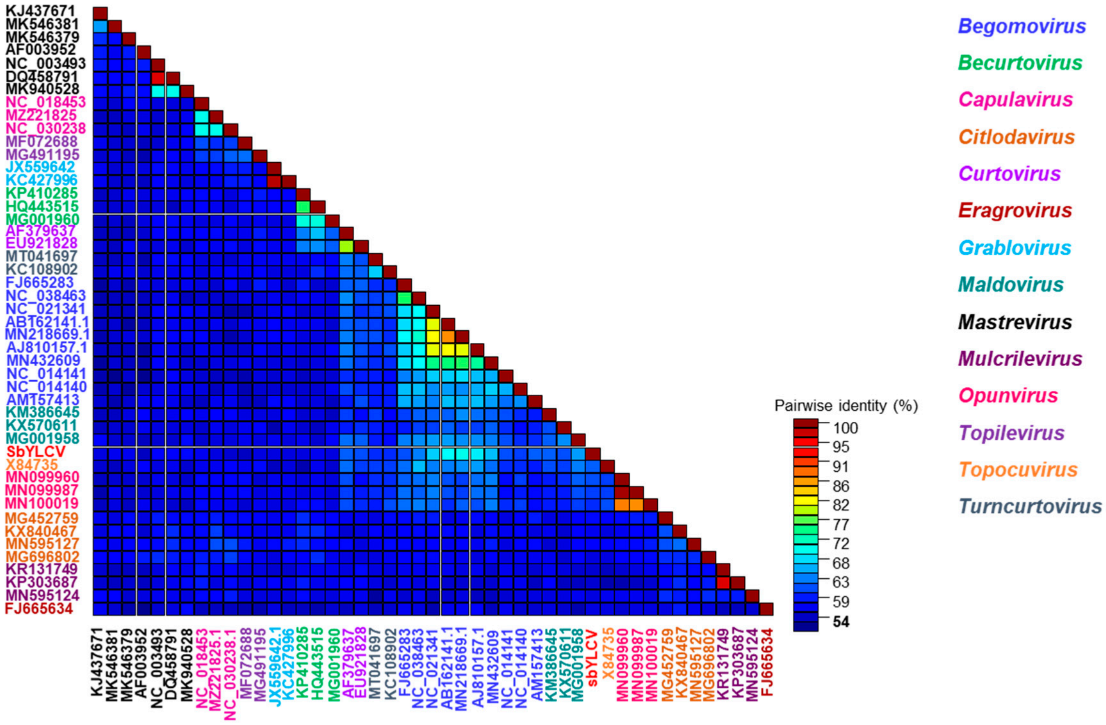

Figure S2. Matrix plot showing the pairwise identity of the amino acid sequences of SbYLCV CP and the CP of representative geminiviruses using the SDT software.

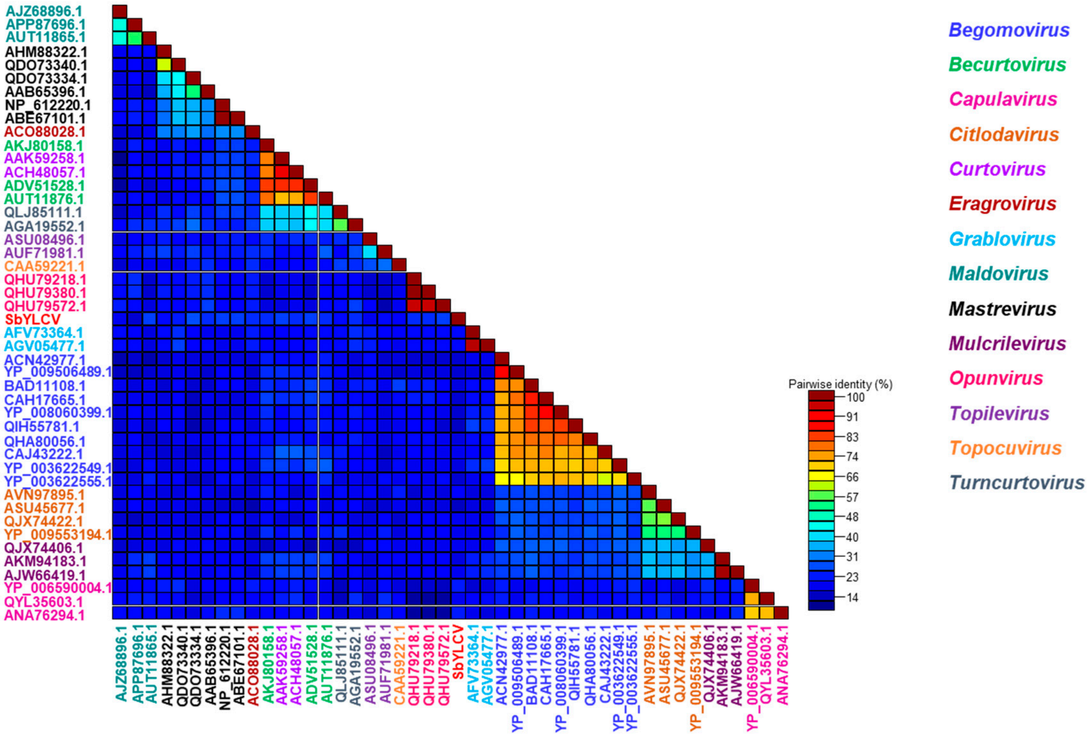

Figure S3. Neighbor-joining phylogeny tree constructed based on the amino acid sequences of SbYLCV Rep and the Rep of the representative geminiviruses. The statistical significance of the branches was estimated with a bootstrap of 1000 replicates.

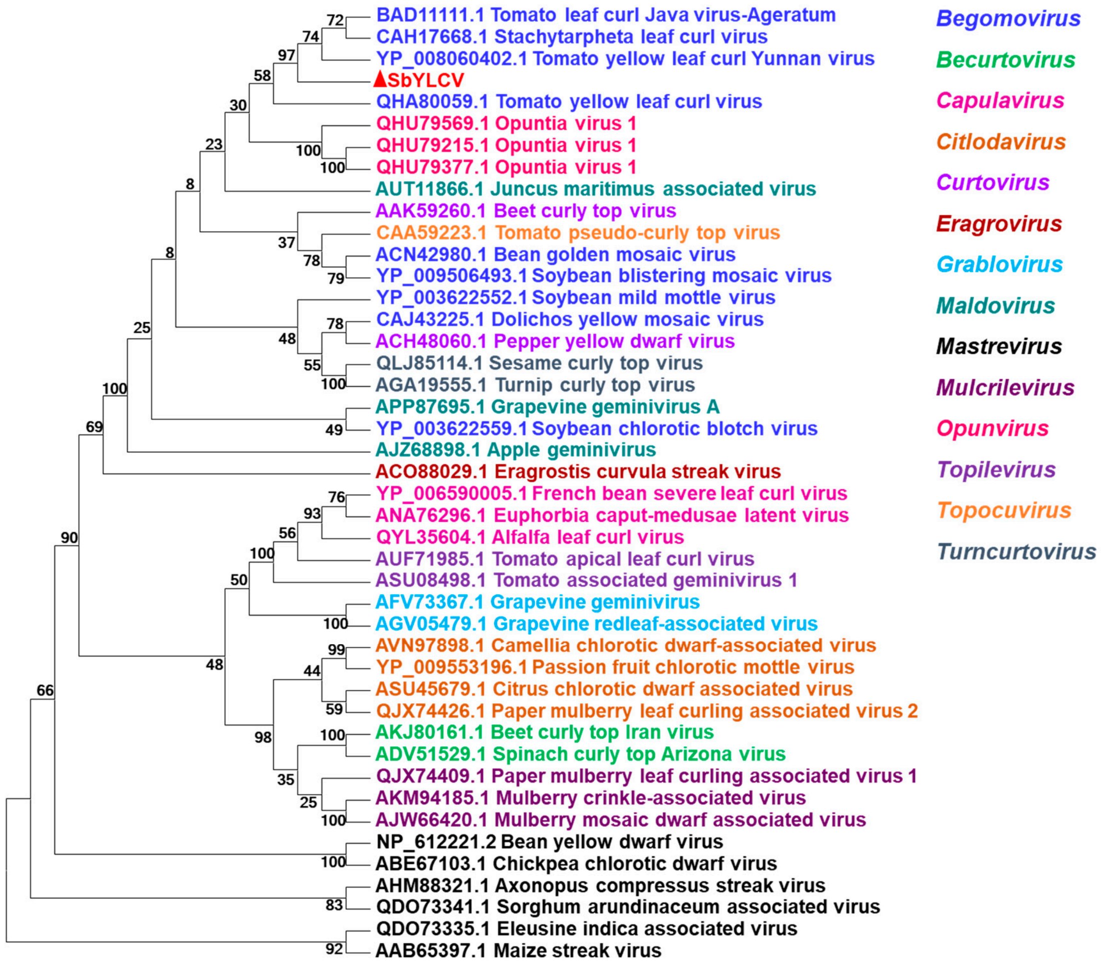

Figure S4. Matrix plot showing the pairwise identity of the amino acid sequences of SbYLCV Rep and the Rep of representative geminiviruses.

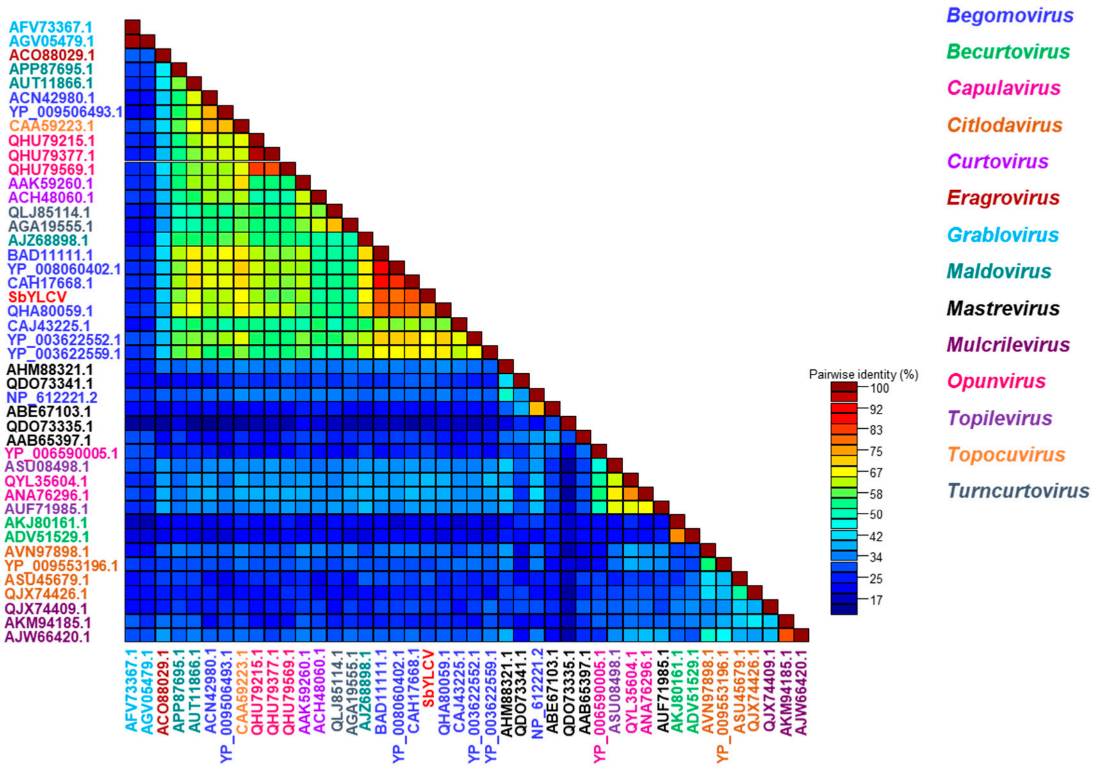

Supplement: Supplementary file 1 [file viruses-14-00341-s001.zip › viruses-1564504-supplementary.pdf]
